# The relationship between traumatic childbirth and first-time mothers’ social identity and wellbeing: a cross-sectional observational study

**DOI:** 10.1186/s12884-024-06288-3

**Published:** 2024-06-21

**Authors:** Shama El-Salahi, Rebecca Knowles Bevis, Lorna Hogg

**Affiliations:** 1 Oxford Health NHS Foundation Trust, Warneford Hospital, Warneford Lane, OX3 7JX Oxford, UK; 2 Oxford Institute of Clinical Psychology Training and Research, Isis Education Centre, University of Oxford, Warneford Hospital, Warneford Lane, OX3 7JX Oxford, UK

**Keywords:** Traumatic childbirth, First-time mothers, Social identity, Wellbeing, Postnatal

## Abstract

**Background:**

Experiencing childbirth as traumatic is common and can have long-lasting negative consequences for women’s mental health. However, fostering a sense of social identity has been shown to protect psychological wellbeing and mental health during life transitions, such as entering parenthood. This study therefore investigated the relationship between traumatic childbirth and first-time mothers’ social identity and their psychological wellbeing, and more specifically whether strength of identity as a first-time mother protected psychological wellbeing following traumatic childbirth.

**Method:**

Women over the age of 18 who were living in the UK and had given birth to their first child in the past nine months were recruited to the study from clinical and community settings. They completed digital self-report questionnaires about their birth experience, social identity, mental health, and psychological wellbeing. Women who perceived themselves to have had a traumatic birth (the trauma group; *N* = 84) were compared to women who did not perceive themselves to have had a traumatic birth (the control group, *N* = 39). T-tests and chi square tests assessed preliminary group differences before multivariate analyses of covariance controlled for covariates. Post-hoc tests identified the direction of differences. Multiple regression and moderation analyses analysed interaction effects.

**Results:**

The trauma group had significantly lower psychological wellbeing (mean = 41.5, 95% CI [39.4–43.7], *p* = .008, partial η^2^ = 0.059), compared to the control group (mean = 48.4, 95% CI [45.3–51.5]), but the two groups did not differ in the strength of their first-time mother identity, which was high across both groups. Strength of identity did not moderate the relationship between traumatic childbirth and psychological wellbeing. Giving birth by caesarean section independently reduced the strength of the first-time mother identity (*p* = .017, partial η^2^ = 0.049). All analyses controlled for emotional and practical support, perceptions of healthcare staff, and mode of birth.

**Conclusions:**

Having a traumatic birth was associated with lower psychological wellbeing, and the strength of first-time mother identity does not appear to moderate this relationship. Factors such as mode of birth may be more important. Further research, including longitudinal designs, is needed to understand the relationship between these constructs and identify more effective ways of protecting first-time mothers’ mental health.

## Background

Childbirth is often a positive, life-enhancing experience, yet as many as one in two women find it traumatic and a significant minority go on to develop postnatal posttraumatic stress disorder (PTSD) [1–3]. Although obstetric complications can increase the risk of PTSD, research suggests that individual perceptions play a more crucial role [4]. Feeling empowered or powerless, and the extent to which women have a sense of trust and control and feel informed during labour, have been found to distinguish between whether they find birth traumatic or not [5]. Postnatally, experiencing birth as traumatic can have far-reaching negative consequences for women [6–8]. Fenech et al.’s (2014) meta-synthesis of the qualitative evidence identified three overarching effects: strong negative emotions and dysfunctional coping strategies, an embodied sense of loss of self and family ideals, and shattered relationships [9]. These themes highlight the relevance of identity and social relationships to the experience of traumatic childbirth. Whilst several terms exist in the literature for traumatic childbirth, a recent concept analysis paper defined it as “the woman’s subjective feeling caused by events directly or indirectly related to childbirth, which is manifested as intertwined painful emotional experiences that originate in the birth process and last until postpartum” (p.11) [10].

For mothers having their first child, traumatic childbirth occurs when they are already contending with the effects of a major life transition - a period involving significant change and adjustment when mental health and social connections are especially vulnerable [11]. Not only must mothers adapt to changed routines, sleep patterns and responsibilities, they must also renegotiate the social identities they hold. Social identity is conceptualised as the sense of self that people derive from membership of a social group [12]. Group memberships are a key source of social support and have a range of benefits for mental health, such as instilling a sense of personal control, meaning, coping and resilience [13–16]. During a major life change, certain social identities may be threatened or lost, and connectedness to groups is critical to the renegotiation of identity and maintenance of good psychological wellbeing [17].

Research demonstrates that fostering a sense of social identity can protect psychological wellbeing during transitions. The Social Identity Model of Identity Change (SIMIC) posits that life transitions weaken our social identities through loss of contact with social groups, leading to poorer wellbeing and mental health [17]. It also suggests that the stressful impact of life transitions can be counteracted by maintaining pre-existing social identities as well as taking on new identities consistent with the life change, and by the compatibility between pre-existing and new identities. There is good evidence for SIMIC across different life transitions [18–20], including within the perinatal field [21, 22].

To the authors’ knowledge, research has not yet explored social identity in relation to traumatic childbirth. This study aims to address this gap in the literature by examining the relationships between traumatic childbirth, strength of the first-time mother identity and psychological wellbeing. Considering that previous literature suggests that traumatic childbirth negatively affects women’s postnatal mental health and maternal experiences [23], and the fact that childbirth precedes postnatal outcomes chronologically, directional hypotheses were predicted. In line with SIMIC and the literature discussed above, three hypotheses were tested:

1. First-time mothers who have had a traumatic birth will have weaker identities as first-time mothers compared to first-time mothers who did not have a traumatic birth.
2. First-time mothers who have had a traumatic birth will have lower levels of postnatal psychological wellbeing than first-time mothers who did not have a traumatic birth.
3. The first-time mother social identity will moderate the relationship between traumatic childbirth and postnatal psychological wellbeing, such that when the first-time mother identity is stronger, the effect of having a traumatic birth on psychological wellbeing will be weaker.

Given that previous research has highlighted the need to identify factors contributing to childbirth being traumatic [5, 10], this study also aimed to collect brief qualitative data on women’s appraisals. This was not integral to the study design, but was included in order to be able to describe the characteristics of traumatic childbirth within the sample and to contextualise the quantitative findings.

## Methods

A cross-sectional between-groups design compared women who had a traumatic birth (trauma group) to women who did not have a traumatic birth (control group) to explore potential differences in social identity and psychological wellbeing. Having read an information sheet about the study, consenting participants completed an anonymous online survey using Qualtrics software. Participants’ names and contact details were not collected, so informed consent was implied through voluntary completion of the questionnaires. Signposting information about accessing mental health support was provided on each page of the survey. The participant information sheet made it clear that it could be distressing to think about birth experiences and encouraged participants to consider carefully whether to take part and to speak to their GP, health visitor, or perinatal mental health team if they were unsure. Participants were also encouraged to contact the researchers if they had any concerns about the study or had been adversely affected by any aspect of it. During the study planning phase, women with lived experience of traumatic childbirth were involved in decisions about the design, procedures, and materials to ensure it was conducted sensitively. The study was approved by a Research Ethics Committee confirming that ethical standards were met (see the Declarations section for more information). Recruitment and data collection ran from October 2020 to March 2021.

### Participants

Women were eligible to take part if they lived in the United Kingdom, were over 18 years of age, and had given birth to their first child within the past nine months to capture the transition to motherhood where maternal identity changes are likely to be most notable. Women who were unable to provide consent or complete questionnaires in English, or whose child died before, during, or after birth or had significant life-threatening illnesses were not eligible to take part as these experiences were thought to be significantly different to the experience of traumatic childbirth alone. Participants were recruited using opportunity sampling from three NHS mental health services who shared an advert about the study signposting potential participants to the survey. The research was also advertised in community settings via social media and birth-related charities. The researchers planned to put posters advertising the study in community locations, such as venues hosting mother-and-baby groups, but this was not possible due to restrictions imposed by the Covid-19 pandemic.

The study aimed to recruit 82 participants based on a priori power calculations using G*Power software [24] and allowing for 20% attrition. No power calculation is available for MANCOVA, and the researchers therefore based the calculation on the multivariate analyses of variance (MANOVA) test, an approach adopted by other researchers [25, 26] and recommended by Dattalo (2008), who suggests adjusting the sample size estimation method by adding the number of covariates in the design to the number of groups [27]. This increased the required sample size by two participants. Statistical power was set at 0.80 and a medium effect size of *f²*=0.15 was chosen, in line with Cohen (1977; 1988) [28, 29]. Regression analyses required a sample of 55 participants based on one predictor variable and an effect size of *f²*=0.15.

### Measures

Self-report questionnaires were administered (see Table 1). Primary measures included exposure to traumatic childbirth, strength of the first-time mother social identity, and psychological wellbeing, whilst the secondary measures included demographic information, postnatal PTSD, postnatal depression, maintenance and compatibility of group memberships during life transitions, and risk and vulnerability factors associated with postnatal PTSD. The primary measures tested the research hypotheses, and the secondary measures allowed the researchers to describe the sample and control for confounding variables. Except for the multiple identity questionnaire (where participants could enter up to six affiliated social groups), participants were required to complete every question to proceed with the survey which minimised missing data. If participants did not enter any social groups on the multiple identity questionnaire, the number of groups was coded as missing data, rather than assuming no social group affiliations.Women who indicated having experienced traumatic childbirth were asked to provide details on the nature of the trauma, factors that contributed most to it being traumatic, and what specifically they found traumatic about childbirth to identify trauma-related attributions. A list of factors that commonly contribute to childbirth being traumatic was presented, which had been developed in collaboration with women with lived experience of traumatic childbirth. Participants were asked to select those that contributed to their experience being traumatic and then to choose the top three that contributed most strongly. Finally, they were asked to elaborate on the reasons why childbirth was traumatic for them, if they felt comfortable to do so.

**Table 1:** Study outcome measures

Domain	Measures	Cronbach’s alpha
**Primary outcome variables**		
Exposure to traumatic birth (independent variable)	Dichotomous question of whether childbirth (or the events leading up to or shortly following birth) was traumatic. Participants who indicated their childbirth was traumatic described the nature of the trauma and then chose three factors that contributed most to childbirth being traumatic. A free-text box allowed them to elaborate on what they found traumatic about childbirth if they felt comfortable doing so.	*N/A*
Strength of first-time mother identity (dependent variable)	The in-group identification questionnaire^1^ is a valid and reliable 14-item scale of in-group identification adapted for this study to be relevant to first-time mothers. Items were scored on a seven-point Likert scale (1 = strongly disagree to 7 = strongly agree) with total scores ranging from 14–98. Higher scores represent stronger identities as first-time mothers.	0.91
Psychological wellbeing (dependent variable)	The Warwick Edinburgh Mental Well-being Scale^2^ (WEMWBS) is a widely used, valid and reliable measure comprised of 14 items scored on a five-point Likert scale (1=‘none of the time’ to 5=‘all of the time’). Total scores range from 14–70 with higher scores representing higher mental wellbeing. Scores between 45–59 represent average wellbeing.	0.94
**Secondary outcome measures**		
Demographics	Participants provided their age, sexuality, ethnicity, employment status over the past 12 months, postcode, marital status, and their baby’s age and health status. Postcodes were used as a rough estimate of the socioeconomic status of the area in which participants lived. Postcodes were converted into a deprivation decile using the governments’ Indices of Multiple Deprivation for each country in the UK and then deleted, thereby de-identifying the data. As the researchers only wanted a rough estimate of socioeconomic status in order to describe the sample, the deciles were transformed into a dichotomous variable where the most deprived 50% of neighbourhoods were compared to the least deprived 50% of neighbourhoods nationally.	*N/A*
Postnatal PTSD	The City Birth Trauma Scale^3^ has 29 items, with total scores ranging from 0–60 with higher scores representing higher symptom severity. This measure was used in two ways: (1) the total score provided the severity of postnatal PTSD symptomatology, and (2) participants’ scores were transformed into a dichotomous variable based on whether or not they met diagnostic criteria for postnatal PTSD according to DSM-V criteria^4^.	0.94
Postnatal depression	The Edinburgh Postnatal Depression Scale^5^ has 10 items and is a widely used, valid and reliable screening tool for postnatal depression. Scores range from 0–30 with higher scores corresponding to increasing symptom severity. Participants scoring 13 or above are likely to be suffering from major postnatal depression^5^. This measure was used in two ways: (1) the total score provided the severity of postnatal depression symptomatology, and (2) participants’ scores were transformed into a dichotomous variable based on whether or not they scored 13 or over and were likely to be suffering from major postnatal depression.	0.89
Maintenance of group memberships during life transitions	The multiple-identity scale^6^ measured changes to group memberships from before to after giving birth. Participants listed up to six social groups they identified with before and after they gave birth. Each group was rated for pre-birth and post-birth importance on a 1–7 Likert scale. Pre-birth compatibility with the other social groups was rated on a 1–7 Likert scale, and post-birth compatibility with the first-time mother identity was rated on the same scale. Higher scores indicated greater group importance and compatibility.	0.99
Risk and vulnerability factors	Previous research has identified a number of risk and vulnerability factors most strongly associated with the development of postnatal PTSD^7^. These include previous psychological problems, history of trauma, fear of childbirth, poor health or complications in pregnancy, type of birth, support during pregnancy, and past treatment/help-seeking for psychological problems. Participants in this study selected which type of birth they had (vaginal/assisted/caesarean) and then selected whether or not they had experienced the other risk and vulnerability factors.	*N/A*

^1^Leach et al. (2008) [30]; ^2^Stewart-Brown et al. (2011) [31]; ^3^Ayers, Wright and Thornton ( 2018) [32]; ^4^American Psychiatric Association (2013) [33]; ^5^Cox, Holden and Sagovsky (1987) [34]; ^6^Haslam et al. (2008) [18]; ^7^Ayers et al. (2016) [35]

### Statistical analyses

For *H*_*1*_ and *H*_*2*_, t-tests and chi square tests ascertained preliminary group differences before multivariate analyses of covariance (MANCOVA) were run to control for covariates. Post-hoc tests identified the direction of differences. For *H*_*3*,_ multiple regression and moderation analyses examined interaction effects. All test assumptions were checked first, and the analyses were conducted in SPSS v27. Reasons for childbirth being traumatic were not formally analysed as they were intended to provide further understanding of women’s experiences, rather than being a key part of the study design. The reasons provided were examined by the first author and themes were extracted.

## Results

### Descriptive statistics

Three hundred and twenty one people accessed the study survey. Of these, 124 participants (38.6%) completed the survey. One participant’s baby was over nine months old, so their data was excluded, leaving a sample of 123 participants (*N* = 84 trauma condition, *N* = 39 control condition). Table 2 locates the sample in its demographic context and Table 3 provides descriptive data on the dependent and secondary outcome variables.

**Table 2:** Demographics of the sample

Variable		Trauma condition (*N* = 84)		Control condition (*N* = 39)		
		Range (min-max)	Mean (SD)		Range (min-max)	Mean (SD)
Age (years)		20–40	30.86 (4.22)		19–39	30.92 (4.47)
Baby’s age (months)		0–9	4.96 (2.30)		0–9	4.59 (2.44)
		***N***	***%***		***N***	***%***
Number of women whose babies had health conditions		6	7.1		4	10.3
Ethnicity	Indian	3	3.6		0	0
	Bangladeshi	0	0		0	0
	Pakistani	0	0		0	0
	Chinese	0	0		0	0
	Other Asian background	0	0		0	0
	Black African	0	0		0	0
	Black Caribbean	0	0		0	0
	Other Black background	0	0		0	0
	White British	70	83.3		37	94.9
	White Irish	3	3.6		0	0
	Other White background	4	4.8		2	5.1
	Mixed White & Asian	0	0		0	0
	Mixed White & Black African	0	0		0	0
	Mixed White & Black Caribbean	1	1.2		0	0
	Other Mixed background	2	2.4		0	0
	Any Other	0	0		0	0
	Prefer not to say	1	1.2		0	0
Sexuality	Heterosexual	78	92.9		38	97.4
	Gay or lesbian	0	0		0	0
	Bisexual	3	3.6		1	2.6
	Other	0	0		0	0
	Prefer not to say	3	3.6		0	0
Work status over past 12 months	Unemployed	2	2.4		0	0
	Employed	67	79.8		33	84.6
	Retired	0	0		0	0
	Homemaker	1	1.2		1	2.6
	Student	0	0		2	5.1
	Non-paid work	0	0		0	0
	Self-employed	7	8.3		3	7.7
	Non-government employee	1	1.2		0	0
	Government employee	4	4.8		0	0
	Prefer not to say	2	2.4		0	0
Marital status	Married	51	60.7		28	71.8
	Living as a couple	29	34.5		11	28.2
	Divorced or separated	0	0		0	0
	Single	1	1.2		0	0
	Widowed	0	0		0	0
	Other	0	0		0	0
	Prefer not to say	3	36		0	0
Deprivation decile	Most deprived 50% neighbourhoods nationally	22	26.2		9	23.1
	Least deprived 50% of neighbourhoods nationally	62	73.8		30	76.9

Note. The trauma condition contained participants who reported experiencing a traumatic birth, whereas the control condition contained participants who did not report experiencing a traumatic birth

**Table 3:** Descriptive data of the dependent and secondary outcome variables

Variable	Trauma condition		Control condition	
	N	Mean (SD)	N	Mean (SD)
Strength of first-time mother identity	84	71.81 (14.62)	39	78.36 (9.59)
Psychological wellbeing	84	41.55 (9.92)	39	48.41 (9.49)
Postnatal PTSD score	84	24.67 (14.17)	39	8.97 (8.79)
Postnatal depression score	84	11.61 (6.00)	39	8.03 (5.75)
Number of social groups before birth	43	2.33 (1.27)	28	2.57 (1.29)
Number of social groups after birth	49	2.55 (1.36)	28	2.75 (1.58)
Group importance before birth	67	3.98 (1.93)	36	3.87 (1.96)
Group importance after birth	58	4.56 (1.81)	30	4.74 (1.81)
Group compatibility before birth	52	3.52 (1.89)	33	3.92 (1.82)
Group compatibility after birth	51	4.98 (1.77)	27	5.13 (1.80)
		***N (%)***		***N (%)***
Unassisted vaginal birth	84	21 (25%)	39	32 (82.1%)
Assisted birth (e.g., forceps)	84	35 (41.7%)	39	3 (7.7%)
Caesarean section	84	28 (33.3%)	39	4 (10.3%)
Felt they received adequate emotional support from staff	84	42 (50%)	39	29 (74.4%)
Felt they received adequate practical support from staff	84	58 (69%)	39	31 (79.5%)
Felt they received adequate emotional support from others	84	71 (84.5%)	39	38 (97.4%)
Felt they received adequate practical support from others	84	65 (77.4%)	39	36 (92.3%)
Felt listened to and included in making decisions related to birth	84	46 (54.8%)	39	35 (89.7%)
Felt staff were kind and attentive	84	56 (66.7%)	39	35 (89.7%)
Had a strong fear of childbirth before birth	84	20 (23.8%)	39	9 (23.1%)
Poor health/complications in pregnancy	84	21 (25%)	39	9 (23.1%)
Pre-existing mental health difficulties	84	21 (25%)	39	11 (28.2%)
Previous exposure to trauma	84	12 (14.3%)	39	8 (20.5%)
Previously received professional support for mental health	84	29 (34.5%)	39	14 (35.9%)
Currently receiving professional support for mental health	84	12 (14.3%)	39	4 (10.3%)
Current medical use for mental health	84	9 (10.7%)	39	2 (5.1%)
Met diagnostic criteria for postnatal PTSD	84	24 (28.6%)	39	0 (0%)
Met screening criteria for major postnatal depression	84	41 (48.8%)	39	8 (20.5%)

Note. The trauma condition contained participants who reported experiencing a traumatic birth, whereas the control condition contained participants who did not report experiencing a traumatic birth

Women in both groups had strong first-time mother identities (trauma group mean = 71.81/98; control group mean = 78.36/98). The trauma group had below average levels of psychological wellbeing (mean = 41.55/70), whilst the control group had average levels (mean = 48.41/70). Loss of blood was rated by the largest number of women as the factor that contributed most to childbirth being traumatic (*N* = 9). Unanticipated separation from a birthing partner was rated as the second strongest factor (*N* = 8) and birth injuries caused to the mother, such as episiotomy and perineal tears, was the third strongest factor (*N* = 7).

### Reasons for childbirth being traumatic

Women who experienced traumatic childbirth described qualitatively why it was traumatic. This information was not formally analysed but instead screened for common themes. Women frequently reported it being traumatic due to a long, painful labour associated with severe blood loss. Many had a high level of medical input including caesarean sections and feared they or their baby would die. A repeated concern was that these experiences interrupted mother-and-baby bonding, and several women talked about feeling as though they had failed or were responsible for difficulties encountered. A lack of communication and support from staff led some women to feel abandoned, not listened to, and that procedures were ‘done to’ them without being informed or included in decisions. Several women reported feeling let down and dismissed by healthcare professionals, particularly when staff did not help to facilitate the relationship with their baby. Many women also shared that the reality of their birth experience was very different to their birth plan, which seems to have been exacerbated by the Covid-19 pandemic’s restrictions on birthing partners being allowed into hospital.

### Preliminary between-group differences

The data met the assumptions for parametric tests. Before controlling for covariates, the trauma group had a significantly weaker first-time mother identity (*t*(107.15) = 2.96, *p* = .004, *d* = 0.68), lower psychological wellbeing (*t*(121) = 3.62, *p* < .001, *d* = 0.72), and higher severity of postnatal PTSD (*t*(110.98)=-7.51, *p* < .001, *d* = 1.78) and depression (*t*(121)=-3.12, *p* = .002, *d* = 0.62), when compared to the control group. The trauma group was less likely to have an unassisted vaginal birth (*X*^*2*^(2, *N* = 123) = 35.52, *p* < .001, Cramer’s *V* = 0.54), and less likely to feel they received adequate emotional support from staff (*X*^*2*^(1, *N* = 123) = 6.48, *p* = .018, Cramer’s *V* = 0.23) or other people (*p* = .037, Fisher’s exact test), or adequate practical support from other people (*X*^*2*^(1, *N* = 123) = 4.04, *p* = .074, *V* = 0.18). They were also less likely to feel listened to and included in decision-making about their birth (*X*^*2*^(1, *N* = 123) = 14.49, *p* < .001, *V* = 0.34) or to feel that staff were kind and attentive to their needs (*X*^*2*^(1, *N* = 123) = 7.37, *p* = .007, *V* = 0.25).

Correlational analyses identified several secondary outcome measures that significantly correlated with the dependent variables (*p* < .05). It would be unfeasible to include this many covariates in the MANCOVA, and the threshold for significance was therefore decreased to *p* < .001 to exclude any less relevant variables (see Table 4). On this basis, five variables were included as covariates: receiving adequate emotional support from staff (*r(121)* = 0.32, *p* < .001), receiving adequate emotional support from other people (*r(121)* = 0.37, *p* < .001), receiving adequate practical support from other people (*r(121)* = 0.30, *p* < .001), feeling staff were kind and attentive to needs (*r(121)* = 0.33, *p* < .001), and giving birth by caesarean section (*r(121)*=-0.38, *p* < .001). The postnatal PTSD and depression variables also significantly correlated with the dependent variables (*p* < .001), but they were not included as covariates as they were considered too conceptually similar to the independent variable (exposure to traumatic childbirth) such that their inclusion would likely cancel out any variance associated with group membership.

**Table 4:** Variables excluded as covariates

Dependent variable	Secondary outcome variable	r	p
Strength of first-time mother identity	Age	− 0.180	0.047
	Received adequate practical support from staff	0.251	0.005
	Felt listened to and included in decision-making related to birth experience	0.266	0.003
	Vaginal birth	0.242	0.007
	Heterosexual	0.245	0.006
	Bisexual	− 0.216	0.016
	Government employee	− 0.216	0.016
	Postnatal PTSD score	− 0.51	*p* < .001
	Postnatal PTSD diagnostic criteria met	− 0.42	*p* < .001
	Postnatal depression score Postnatal depression screening cut-off met	− 0.51	*p* < .001
		− 0.37	*p* < .001
Psychological wellbeing	Felt listened to and included in decision-making related to birth experience	0.241	0.007
	Strong fear of childbirth before giving birth	− 0.199	0.028
	Currently taking medication for mental health	− 0.195	0.031
	Number of social groups before birth	0.294	0.013
	Mean group importance before birth	− 0.233	0.018
	Postnatal PTSD score	− 0.69	*p* < .001
	Postnatal PTSD diagnostic criteria met	− 0.52	*p* < .001
	Postnatal depression score Postnatal depression screening cut-off met	− 0.82	*p* < .001
		− 0.66	*p* < .001

### Tests of hypotheses

There were statistically significant between-group differences on the combined dependent variables after controlling for covariates (*F*(2, 115) = 3.876, *p* = .024, Wilk’s Λ = 0.937, partial η^2^ = 0.063). Giving birth by caesarean section was the only factor that significantly influenced strength of the first-time mother social identity and psychological wellbeing after controlling for the effects of all other variables (*F*(2,115) = 5.260, *p* = .007, Wilk’s Λ = 0.916, partial η^2^ = 0.084). Post-hoc power analyses of both the MANOVA and ANCOVA showed that statistical power of the study was 0.67–0.79, taking into account the unequal group sizes. Box’s test for equality of covariance was significant (*p* = .031) but as the MANCOVA is robust [36] this violation was not deemed to significantly affect the test results.

#### Traumatic childbirth and strength of first-time mother identity (H_1_)

Between-subjects comparisons showed that exposure to traumatic childbirth did not have a significant main effect on strength of the first-time mother identity (*F*(1,116) = 0.668, *p* = .416, partial η^2^ = 0.006). However, having a caesarean section did, with a medium effect size (*F*(1,116) = 5.914, *p* = .017, partial η^2^ = 0.049): comparison of the estimated marginal means showed that overall strength of the first-time mother identity was significantly lower for the group who had a caesarean section (mean = 69.11, standard error = 2.24) compared to the group who had a vaginal birth with or without instrumental assistance (mean = 75.57, standard error = 1.28).

#### Traumatic childbirth and psychological wellbeing (H_2_)

Between-subjects comparisons showed that exposure to traumatic childbirth had a significant main effect of medium magnitude on psychological wellbeing (*F*(1,116) = 7.324, *p* = .008, partial η^2^ = 0.059): comparing the estimated marginal means showed that psychological wellbeing was lower in the trauma group (mean = 42.03, standard error = 1.07) compared to the control group (mean = 47.37, standard error = 1.60). Having a caesarean section had no significant main effect on wellbeing (*F*(1,116) = 0.329, *p* = .567, partial η^2^ = 0.003).

#### The moderating effect of the strength of first-time mother identity on the relationship between traumatic childbirth and psychological wellbeing (H_3_)

From the multiple regression and moderation analyses, the overall model was statistically significant after controlling for the five covariates (*F*(8,114) = 9.544, *p* < .0001, *R*^*2*^ = 0.401). Traumatic childbirth had a significant main effect on psychological wellbeing (*b*=-3.969, *t*(114)=-2.201, *p* = .030), as did strength of first-time mother identity (*b* = 0.530, *t*(114) = 3.667, *p* < .001). Exposure to traumatic childbirth was linked to a 3.969-unit reduction in wellbeing, whilst for every 1-unit increase in strength of identity there was a 0.530-unit increase in wellbeing. The interaction effect of traumatic childbirth and strength of identity on psychological wellbeing was not statistically significant (*b*=-0.137, *t*(114)=-0.857, *p* = .393), meaning that strength of identity as a first-time mother did not moderate the strength of the relationship between traumatic childbirth and psychological wellbeing.

## Discussion

This study investigated the relationship between having a traumatic birth and women’s psychological wellbeing and on their social identity as first-time mothers. Evidence was found only in support of *H*_*2*_: having a traumatic birth resulted in lower levels of psychological wellbeing in first-time mothers, which is consistent with previous research [9]. The data did not support *H*_*1*_ or *H*_*3*_: traumatic childbirth did not appear to be predictive of the strength of the first-time mother identity, and strength of identity did not moderate the observed relationship between traumatic childbirth and psychological wellbeing. Although preliminary tests of group differences revealed that women who had a traumatic birth appeared to have weaker identities as first-time mothers, the effect disappeared when controlling for covariates, suggesting that the effect of traumatic childbirth on the strength of first-time mother identity may be mediated by other variables, such as mode of birth. Having a caesarean section (as opposed to a vaginal or instrumental delivery) emerged as one birth-related variable that did weaken the first-time mother identity. This is consistent with research demonstrating that caesarean sections, and particularly those that are unplanned, are associated with difficulties in maternal identity formation [37, 38]. Previous systematic reviews and meta-analyses have found that emergency caesarean sections are also associated with posttraumatic stress [39, 40], which reinforces the hypothesis that mode of birth connects the fields of birth trauma and maternal identity.

Qualitative data provided some insight into why caesarean sections may weaken first-time mothers’ identity. Birth-related injuries (often linked to having a caesarean section) were identified as limiting opportunities for mother-and-baby bonding due to reduced physical strength and mobility. Additionally, staff were often perceived as unsupportive when women could not care for their babies independently due to their physical limitations or injuries, leading to negative views of the quality of care received. Poor-quality interactions with healthcare providers have been extensively documented in the maternity literature and identified as a significant risk factor for the development of clinically-relevant symptoms of postnatal PTSD [41–44]. Healthcare providers supporting their staff to facilitate mother-and-baby connections, particularly after caesarean birth, is an important approach to consider in improving maternal wellbeing and reducing the risk of later psychopathology.

The study findings do not provide clear support for SIMIC, which is surprising considering the wider literature suggesting social identities can buffer the negative effects of life transitions and help to maintain health and wellbeing [18–20, 22]. This may point towards the complexity of maternity identity development and possible ceiling effects of the social identity measure, whereby there was not enough variance in scores to pick up between-group differences; perhaps the in-group identification measure used in this study was not a sufficiently nuanced measure. There are indeed other factors not considered in this study that affect maternal wellbeing. Epigenetics is an area of increasing interest among researchers and there is accumulating evidence that exposure to pain and stress during pregnancy can lead to epigenetic modifications at the foetal, maternal and placental levels that affect gene expression in mother and baby [45, 46]. Such changes during the perinatal periods have been linked to adverse maternal outcomes including postnatal depression and later life psychopathology for offspring [47–49]. Future interdisciplinary research that includes an understanding of epigenetics would be useful for identifying new ways of protecting women’s mental health and that of their children.

### Limitations

There are important limitations to consider when interpreting these findings. First, the study was conducted in a relatively small area of England during the Covid-19 pandemic and despite efforts to broaden the ethnic and geographical diversity of the sample, most participants identified as White British and lived in less deprived areas. The findings may therefore not generalise to first-time mothers from other backgrounds. This is particularly important considering the detrimental consequences of being from ethnic minority backgrounds on perinatal health in the UK [50]. Second, relevant covariates could have been excluded by reducing the significance threshold in the covariate selection process. Multiple methods exist for covariate selection [51] and a conservative approach was considered sensible despite the recognised risks. Third, the reported rates of traumatic birth were double the rates of non-traumatic birth, which possibly reflects a sampling bias where women who experience a traumatic birth are more likely to participate in a study on the topic, leading to a higher incidence of traumatic childbirth in the study compared to the general population. The rates might also be influenced by the timing of the study as it is widely acknowledged that Covid-19 restrictions had a significant adverse impact on pregnant and birthing women [52]. Fourth, given the cross-sectional design it is not possible to draw causal inferences from the findings. It is possible that poorer wellbeing leads to childbirth being experienced as traumatic, as well as the experience of traumatic childbirth leading to poorer wellbeing. However, the theoretical rationale coupled with the fact that childbirth precedes postnatal outcomes lends weight to the interpretation that experiencing a traumatic birth affects how women cope and feel about themselves as mothers in the postnatal period. Although potential recall bias is possible, women’s perceptions of childbirth have been found to be consistent over time [53–56], therefore the traumatic childbirth perceptions in this study are likely to have been established before social identity and wellbeing were measured. Fifth, the qualitative data was not analysed formally, which may threaten its reliability.

### Conclusions and directions for future research

In summary, this study suggests that the relationship between traumatic childbirth and first-time mothers’ social identity is complex and may have an indirect relationship through specific factors such as mode of birth. The findings reinforce the importance of improving interactions between healthcare providers and first-time mothers as healthcare staff play an important role in women’s birth experiences and later psychopathology. This study offers a novel contribution to the literature by advancing understanding of how traumatic birth experiences relate to first-time mothers’ social identity in the transition to motherhood. However, experimental and longitudinal research designs will be needed to understand the causal relationship between traumatic childbirth, maternal identity and wellbeing. Investigating in more detail the relationships between having a caesarean section and the development of a first-time mother identity may be particularly helpful for finding novel and effective ways of supporting first-time mothers during the perinatal period.

## Data Availability

The dataset generated and analysed during the current study are available in the Oxford University Research Archive repository, https://ora.ox.ac.uk/objects/uuid:1f97171d-e57f-4496-afc6-d6c83af1ac44 [57].
